# Carbon-dependent control of electron transfer and central carbon pathway genes for methane biosynthesis in the Archaean, *Methanosarcina acetivorans *strain C2A

**DOI:** 10.1186/1471-2180-10-62

**Published:** 2010-02-23

**Authors:** Lars Rohlin, Robert P Gunsalus

**Affiliations:** 1 Department of Microbiology Immunology and Molecular Genetics, University of California, Los Angeles, 405 Hilgard Avenue, Los Angeles, CA 90095, USA; 2 Molecular Biology Institute, University of California, Los Angeles, 405 Hilgard Avenue, Los Angeles, CA 90095, USA

## Abstract

**Background:**

The archaeon, *Methanosarcina acetivorans *strain C2A forms methane, a potent greenhouse gas, from a variety of one-carbon substrates and acetate. Whereas the biochemical pathways leading to methane formation are well understood, little is known about the expression of the many of the genes that encode proteins needed for carbon flow, electron transfer and/or energy conservation. Quantitative transcript analysis was performed on twenty gene clusters encompassing over one hundred genes in *M. acetivorans *that encode enzymes/proteins with known or potential roles in substrate conversion to methane.

**Results:**

The expression of many seemingly "redundant" genes/gene clusters establish substrate dependent control of approximately seventy genes for methane production by the pathways for methanol and acetate utilization. These include genes for soluble-type and membrane-type heterodisulfide reductases (*hdr*), hydrogenases including genes for a *vht*-type F420 non-reducing hydrogenase, molybdenum-type (*fmd*) as well as tungsten-type (*fwd*) formylmethanofuran dehydrogenases, genes for *rnf *and *mrp-*type electron transfer complexes, for acetate uptake, plus multiple genes for *aha- *and *atp*-type ATP synthesis complexes. Analysis of promoters for seven gene clusters reveal UTR leaders of 51-137 nucleotides in length, raising the possibility of both transcriptional and translational levels of control.

**Conclusions:**

The above findings establish the differential and coordinated expression of two major gene families in *M. acetivorans *in response to carbon/energy supply. Furthermore, the quantitative mRNA measurements demonstrate the dynamic range for modulating transcript abundance. Since many of these gene clusters in *M. acetivorans *are also present in other *Methanosarcina *species including *M. mazei*, and in *M. barkeri*, these findings provide a basis for predicting related control in these environmentally significant methanogens.

## Background

*Methanosarcina acetivorans *strain C2A is a mesophilic anaerobic archaean isolated from a kelp-degrading enrichment of marine origin [1]. It is one of the more metabolically versatile methanogens in that it can use acetate as well as one-carbon substrates including mono-methylamine, di-methylamine, tri-methyl amine, methanol, or carbon monoxide as a sole source of carbon and energy. As a result, it contributes to the formation of two green house gases, methane and carbon dioxide during the natural recycling of organic carbon in anaerobic environments. The biochemical pathways for carbon flow from the alternative substrates to methane are reasonably well established [2-4]. However, little is yet known about the expression of the genes encoding the described pathway enzymes or accessory proteins needed for electron and carbon flow. Additionally, the genome contains seemingly redundant copies of many other genes with implied roles in carbon or energy metabolism [5]. For example, *M. acetivorans *possesses four gene clusters annotated for formylmethanofuran dehydrogenase, three gene sets annotated for hydrogenase, five distinct clusters of genes encoding membrane-bound and/or soluble-type heterodisulfide reductase enzymes, and two gene clusters encoding distinct membrane bound ATP sythase complexes. Orthologs of many of these genes are present in other described *Methanosarcinaceae *species including *M. acetivorans*, *M. mazei*, and *M. barkeri *(Table 1, described below), plus in other methanogenic species.

**Table 1:** Comparison of genes^a ^and corresponding enzyme complexes in sequenced *Methanosarcina *genomes.

Name	***M. acetivorans***	***M. mazei***	***M. barkeri***
*atpDCIXHBEFAG*	Y	N	Y
*ahaHIKECFABD*	Y	Y	Y
*fpoPABCDHIJJKLMNO *operon	Y	Y	Y
*vhtG1A1C1D1*	Y	Y	Y
*vhtG2A2C2*	Y	Y	Y
*frhADGB*	Y	Y	Y
*vhoGAC*	N	Y	N
*echABCDEF*	N	Y	Y
*rnfXCDGEABY*	Y	N	N
*mrpABCDEFG*	Y	N	N
*hdrED1*	Y	Y	Y
*hdrD2*	Y	Y	Y
*hdrA1-pfd*	Y	Y	Y
*hdrC1B1*	Y	Y	Y
*hdrA2B2C2*	Y	Y	Y
*fmdE1F1A1C1D1B1*	Y	Y	Y
*fmdF2A2C2D2B2*	Y	N	N
*fmdB3*	Y	N	Y
*fwdD1B1A1C1*	Y	Y	N
*fwdG2B2D2*	Y	Y	Y
*fwdG1*	Y	Y	N
*fwdE1*	Y	Y	Y
*aceP*	Y	Y	Y
*pta ack*	Y	Y	Y

^**a**^The presence/absence of the corresponding genes/enzymes in the three genomes are indicated by Y (yes) or N (no). For a complete inventory of all *M. acetivorans *genes and designations listed see Figures 1-6.

The expression and/or physiological roles of many of these genes are either poorly understood or unknown. Initial genomic and proteomic studies with *M. acetivorans *and *M. mazei *have initially addressed this but did not clearly resolve these questions due in part to DNA/protein sequence similarities and/or detection limits of the methods used [6]. Additionally, these approaches did not quantitatively address how mRNA abundance levels vary during the alternative cell growth conditions.

In the present study we address the above questions using *M. acetivorans *as a model system to examine gene expression in response to substrate availability. Using quantitative PCR and supporting molecular methods, the resulting data establish expression levels of genes for over twenty enzymes/enzyme complexes for carbon flow and/or energy conservation. The resulting findings define two major substrate-specific gene families for acetate and methanol utilization for this model organism. These studies also lay a foundation to purse the molecular basis of central catabolic pathway gene regulation in this major class of methanogenic archaea.

## Results

### Gene redundancy in the *M. acetivorans *genome

The *M. acetivorans *genome contains many seemingly redundant copies of genes annotated with roles in methanogenesis [5]. These include two sets of genes annotated for a molybdate-type formylmethanofuran dehydrogenase (*fmd*), and two gene sets for a tunsten-type formylmethanofuran dehydrogenase (*fwd*), five heterodisulfide reductase-like *hdrED *and *hdrABC *gene clusters for reduction of Coenzyme M-Coenzyme B heterodisulfide, two sets of *vht *genes for F420 non-reducing hydrogenase, and two sets of genes for ATP synthesizing complexes [5]. Additional genes include *frh *hydrogenase-like genes, plus additional genes for *rnf- *and *mrp*-type membrane associated bacterial electron transfer complexes, plus genes needed for acetate metabolism (discussed below). Homologous and seemingly "redundant" genes/gene sets are also found in the genomes of *M. mazei*, and *M. barkeri *(Table 1). The reason for these genome makeups is currently unknown. *M. acetivorans *was used as a model microorganism to evaluate expression of over twenty sets of genes using gene specific primer pairs designed to eliminate cross-hybridization when DNA sequence similarity exists (Methods). RT-PCR, pPCR, and 5' analysis was then performed using RNA isolated from *M. acetivorans *cells grown with either acetate or methanol as the sole source of carbon and energy. In this study, a number of new *M. acetivorans *gene designations were established to distinguish among homologous orfs (Table 1, and described below).

### Formylmethanofuran dehydrogenase (*fmd, fwd*) gene expression

**Figure 1:**
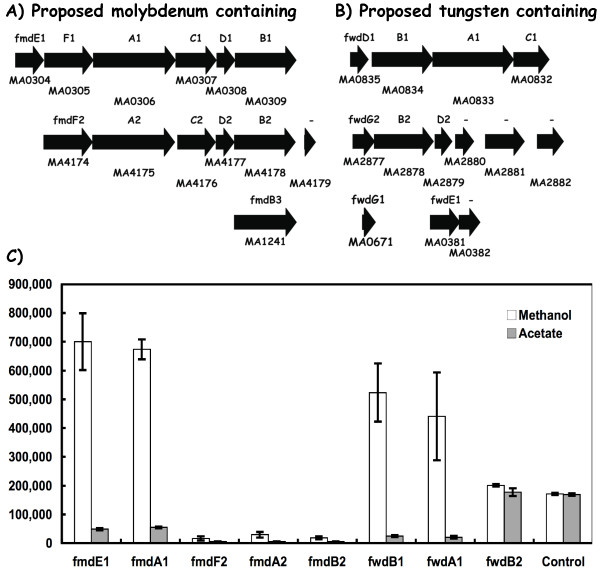
**Differential expression of genes annotated for *fmd *and *fwd *in *M. acetivorans***. Panel A) the six and five gene *fmdE1F1A1C1D1B1 *and *fmdF2A2C2D2B2 *clusters encoding the two putative molybdenum-type formylmethanofuran dehydrogenase enzyme complexes. Panel B) the four and three gene *fwdD1B1A1C1 *and *fwdG2B2D2 *clusters encoding the two putative tungsten-type formylmethanofuran dehydrogenase enzyme complexes. The Genebank identification number (MA number) is shown below each gene while the individual gene designation is shown above. Panel C) RT-PCR data for the indicated *fmd *and *fwd *genes. Values are expressed as copy number (Methods).

Two of the four previously annotated sets of genes for formylmethanofuran dehydrogenasethese were designated as molybdenum-type enzymes and are named here as *fmdE1F1A1C1D1B1 *and *fmdF2A2C2D2B2 *(Figure 1A). Two additional gene sets were annotated as tungsten-type formylmethanofuran dehydrogenase, and are designated here as *fwdD1B1A1C1 *and *fwdG2B2D2 *(Figure 1B). Using qPCR analysis methods (Methods), the molybdenum-type operon reporter genes *fmdE1 *and *fmdA1 *(Figure 1A) were shown to be expressed at 14-fold higher levels during methanol growth conditions relative to acetate growth (Figure 1C). The second set of reporter genes (*fmdF2, fmdA2*, and *fmdB2*) were expressed about 2-fold higher during these conditions, but the maximal level of expression was less than 5% of that seen for the *fmdE1 *and *fmdA1*genes. Noteworthy, the *fmdE1 *and *fmdA1 *gene expression values were within the same range observed for the *fpoN *and *fpoL *genes that encode subunits of the F420 H_2 _dehydrogenase needed for central pathway electron transfer functions (described below). The high transcript abundance of the *fmdE1F1A1C1D1B1 *gene cluster implies a major role of this gene set during methanogenesis in contrast to that for the *fmd2 *gene set.

The annotated tungsten containing formylmethanofuran dehydrogenase gene cluster *fwdD1B1A1C1 *reporter genes designated *fwdB1 *and *fwdA1 *(Figure 1B) were also expressed 15-fold higher levels during methanol growth relative to acetate (Figure 1C). Interestingly, this was within the magnitude observed for the *fmdE1F1A1C1D1B1 *gene cluster. However, the second tungsten-type gene cluster (as reported by the *fwdB2 *gene), was constitutively expressed and at a level about one-half of that observed for either *fwdA1 *or *fwdB1*. These *fmd/fwd *transcript abundance measurements clearly demonstrate that two of the four *fmd *and *fwd *gene clusters (i.e., *fmdE1F1A1C1D1B1 *and *fwdD1B1A1C1*) are highly transcribed in response to substrate availability, and furthermore this suggests that two distinct formylmethanofuran dehydrogenase activities are concurrently utilized during methanol growth conditions (discussed below).

### Heterodisulfide reductase gene expression

**Figure 2:**
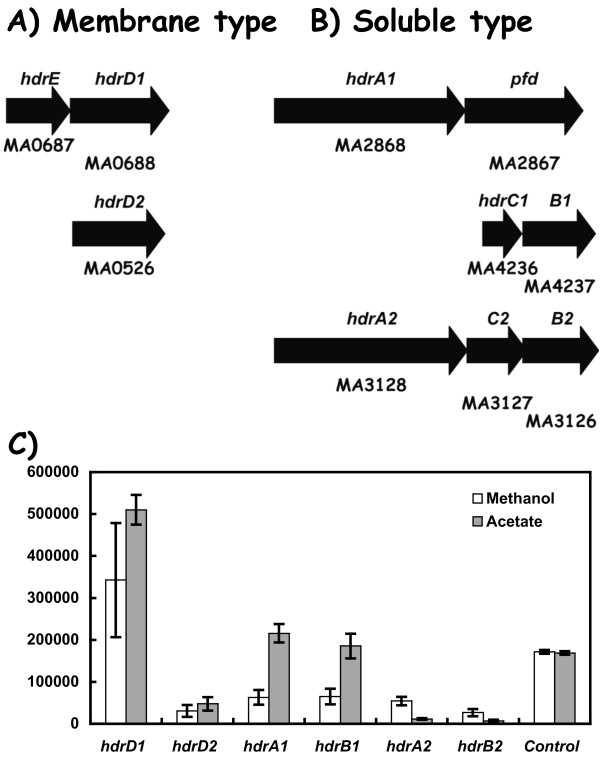
**Differential expression of genes in *M. acetivorans *annotated for *hdr *(hetero-disulfide reductase)**. Panel A) Genes encoding the putative membrane-type hetero-disulfide reductase subunits, *hdrED1 *and *hdrD2*. Panel B) Genes encoding the putative soluble-type hetero-disulfide reductase subunits, *hdrA1 pfd, hdrC1B1*, and *hdrA2C2B2*. The Genebank identification number (MA number) is shown below each gene while the individual gene designation is shown above. Panel C) RT-PCR data for the indicated *hdr *genes.

*M. acetivorans *genome analysis revealed five genes/gene clusters annotated as heterodisulfide reductase, an enzyme essential for electron transfer from methanogenic electron donors to methyl-CoM reductase (Table 1, Figure 2A). These include genes for a membrane-type protein designated here as *hdrE1, hdrD1 *and *hdrD2 *similar to those needed for methane formation in *M. barkeri *[7]. An additional six genes encoding soluble-type heterodisulfide reductase proteins are also present in the genome. They include the *hdrA1 *gene associated with a poly-ferredoxin-like gene (*pfd*), an unlinked set of *hdrCB *genes called *hdrC1*and *hdrB1*, and a third *hdr *gene cluster designated *hdrA2 hdrC2 hdrB2 *(Figure 2B).

Quantitative gene expression experiments (Figure 2C) revealed that the membrane-type *hdrD1 *gene was most highly expressed during acetate cell growth conditions, and where methanol conditions gave slightly lower transcript abundance (ca. 0.7-fold). In contrast, *hdrD2 *gene expression was very low (i.e., at level of about one twentieth that seen for the *hdrD1*gene Figure 2C), suggesting a minor or no direct function in methanogenesis. Interestingly, the abundance of the soluble-type *hdrA1pfd *and *hdrC1B1 *gene transcripts were also nearly as high as for the membrane type *hdrED1 *genes (Figure 2C). Here, acetate growth gave three-fold higher *hdrA1 *transcript levels versus methanol growth conditions. The participation of a soluble-type *hdrABC *enzyme in *M. acetivorans *metabolism is currently unknown but must now be considered. An orf following the *hdrA1 *gene is annotated as a polyferredoxin (*pfd*), and this suggests a role for this protein in electron transfer to couple the soluble-type Hdr complex with an appropriate electron donor complex. In contrast, *hdrA2 *and *hdrB2 *transcript abundance was about two to twenty-fold lower under the corresponding conditions. This suggests a minor role for the second set of HdrABC-type genes (i.e., *hdrA2B2C2*) in methanogenesis.

The *hdrA1pfd *and *hdrC1B1*genes for the soluble-type enzyme subunits are located at different chromosomal loci, and are coordinately expressed since their mRNA abundance levels are alike (Figure 2C). Additionally, the PCR-based gene experiments also demonstrate that the *hdrA1pfd *and the *hdrED1 *genes are each expressed as operons (data not shown). Taken together, these data are consistent with a need for both a membrane-type and a soluble type Hdr enzyme for electron transfer/energy conservation under acetate and methanol cell growth conditions. This suggests that distinct electron transfer pathways are operating to service the alternative Hdr enzymes.

### The *vht *and *frh *gene clusters

**Figure 3:**
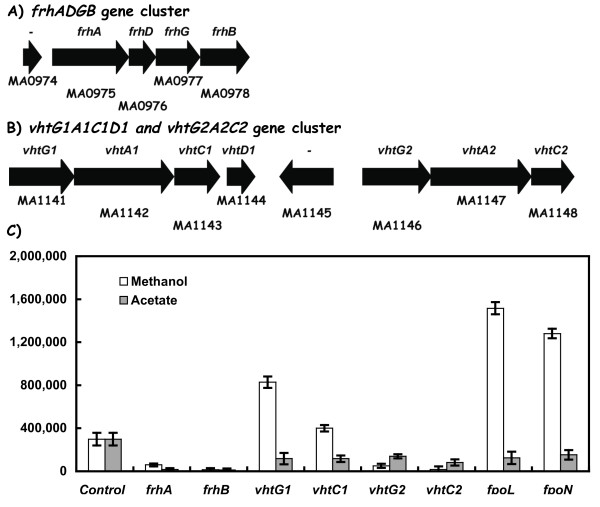
**Differential expression of genes annotated for *vht *(F420 non-reducing hydrogenase) and *frhADGB *(F420 reducing hydrogenase) in *M. acetivorans***. Panel A) The genes encoding the *frhADGB *F420 reducing hydrogenase subunits. Panel B) The genes encoding the *vhtG1A1C1D1 *and the *vhtG2A2C2 *F420 non-reducing hydrogenases. The Genebank identification number (MA number) is shown below each gene while the individual gene designation is shown above. Panel C) RT-PCR data for the indicated genes.

The *M. acetivorans *genome lacks an *echABCDEF *gene cluster encoding an Ech-type hydrogenase with described roles in hydrogen uptake and ion translocation in *M. mazei *[3,5]. Since *M. acetivorans *cells do not exhibit significant hydrogenase activity [8,9], some other mechanism must provide a means for electron transfer from cellular donor(s) to Hdr. Interestingly, the *M. acetivorans *genome contains two sets of genes (designated *vhtG1A1C1D1, and vhtG2A2C2*) for F420-nonreducing hydrogenase-types (Figure 3A, 3B, Table 1). It also contains a set of *frhADGB *genes for a coenzyme F420-type hydrogenase (Figure 3A). Quantitative RT-PCR assays (Figure 3C) established that the *vhtG1 *and *vhtC1*genes were each expressed at four- to six-fold higher levels during methanol growth conditions, and this is within the range seen for the *fpoL *and *fpoN *genes needed for methyl group oxidation for methanol and acetate metabolism. In contrast, expression of the *vhtG2 *and *vhtC2 *genes was low under all conditions examined (Ca. about 17-20-fold lower than *vhtG1 *and *vhtC1*). Finally, the *frhA *and *frhB *gene expression levels were low relative to *vhtG1 *or *fpoL *(Figure 3C), and this suggests a minor role for the *frhADGB *and *vhtG2A2C2 *gene clusters in either methanol or acetate-dependent cell growth. Since *vhtG1 *transcript abundance was elevated and about half of that observed for the *fpoL *and *fpoN *genes that encode subunits of the F420 H_2 _dehydrogenase (Figure 3C), this implies a significant physiological role for the *vhtG1A1C1D1 *gene products during methanol growth. The biochemical and physiological roles for the *vhtG1 *and *vhtC1 *hydrogenase-type genes in *M. acetivorans *are presently unknown.

### The *rnfXCDGEABY *gene cluster is abundantly expressed

**Figure 4:**
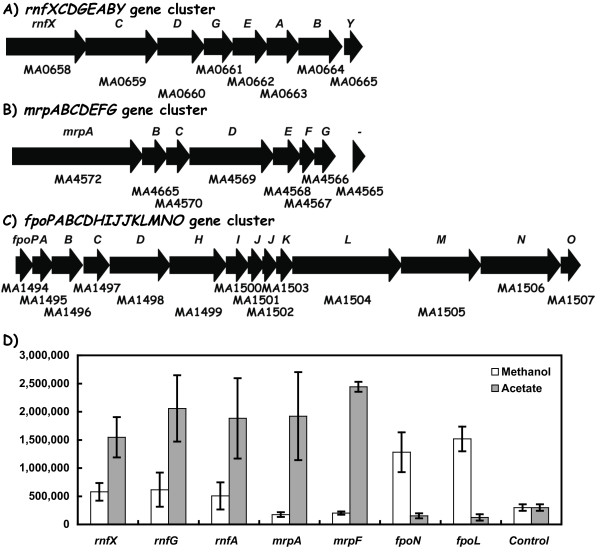
**Differential expression of genes related to electron transport in *M. acetivorans***. The orientation and relative length of each gene is indicated by the open arrows. The Genebank identification number (MA number) is shown below each gene. Panels: A) The eight gene *rnf *cluster; B) the seven gene *mrp *cluster; C) the fourteen gene *fpo *cluster; and D), RT-PCR data for the indicated *rnf, mrp*, and *fpo *genes.

*M. acetivorans *contains a set of six genes (MA0659-0664) annotated as *nqr123456 *[5] that are absent in the *M. mazei*, and *M. barkeri *genomes (Table 1). These genes were subsequently re-designated *rnfCDGEAB *based on sequence comparisons to the *rnf *and *nqr*-type genes in other microorganisms, [10]. This gene cluster also contains two additional genes of unknown function that we designate here as *rnfX *and *rnfY *(Figure 4A) whereby the first (MA0658) precedes *rnfC *and the second (MA0665) follows *rnfB*. We propose that these genes may encode unique input/output modules for membrane associated electron transfer since they are absent in other microbial genomes. During acetate cell growth relative to methanol growth conditions, the *rnfX*, *rnfG*, and *rnfA *reporter genes exhibited elevated transcript abundance (ca. 2.5 to 3.5-fold; Figure 4D). Each gene was also more highly expressed than many reference genes involved in central methanogenesis (e.g., *fpoN*, and *fpoL *that encode subunits of the F420 H2 dehydrogenase). Therefore, the *rnfXCDGEABY *gene expression data support the proposal that the products participate in electron transfer during acetate metabolism as proposed via methanophenazine [10]. In addition, they must also function during methanol culture conditions based on transcript abundance (Figure 4D). Other roles can be envisioned including participation in electron transfer to a soluble-type heterodisulfide reductase via a poly-ferredoxin (e.g., encoded by the *hdrA1 pfd *and *hdrC1B1 *gene complex, described below).

### The *mrpABCDEFG *gene cluster is acetate induced

The *M. acetivorans *genome contains a set of seven genes called *mrpABCDEFG *(Figure 4B) with similarity to the gene clusters found in a variety of bacterial species but absent in either *M. barkeri *or *M. mazei *(Table 1) [5,11-13]. The *mrp-*encoded protein complex in *Bacillus subtilis *was shown to confer a role in multiple drug resistance and/or pH regulation [13,14]. As revealed by the *M. acetivorans *transcript analysis studies (Figure 4D), the *mrpA *and *mrpF *reporter genes were expressed more highly during acetate cell growth conditions (Ca. 11 to 12-fold) relative to methanol growth. These levels were above the expression levels observed for the *ack, pta*, and *hdr *genes needed for acetate utilization, and within the range seen for the *rnf *gene cluster. These findings imply a major role for the six *mrp *gene products in acetate metabolism versus methanol metabolism.

### Expression of the *atp *and *aha *genes encoding ATP synthase complexes

**Figure 5:**
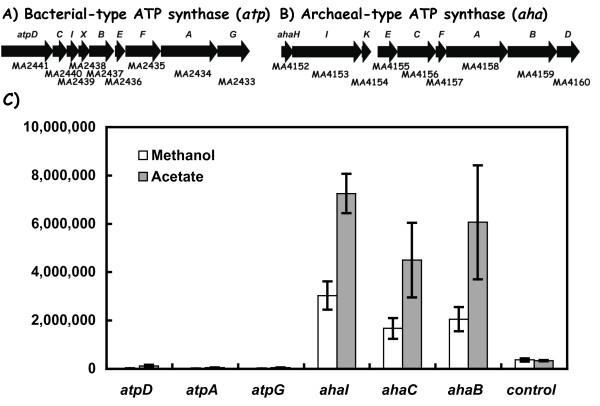
**Expression of the *atpDCIHBEFAG *and the *ahaHIKECFABD *gene clusters encoding the bacterial-type and the archaea-type ATP synthase complexes of *M. acetivorans*, respectively**. The Genebank identification number (MA number), and individual gene designation are shown above or below each gene. Panel C shows RT-PCR data for the indicated *atp *and *aha *gene clusters.

*M. acetivorans *contains genes for a bacterial-type F_0_F_1 _synthase encoded by the MA2441 to MA2433 genes designated here as *atpDCIHBEFAG*, plus an archaeal-type A_0_A_1 _ATP synthase encoded by the *ahaHIKECFABD *genes (MA4152 to MA4160) (Figure 5). Although prior DNA microarray experiments [6] demonstrated that six of the nine genes in the archaeal-type A_0_A_1 _ATP synthase *(ahaECFABD) *encoding the ATP-hydrolysing/synthesizing domain (A_1_) were expressed two-fold higher in acetate grown cells relative to methanol, the other genes were not [6]. It is still unknown how their expression varies quantitatively relative to *atpDCIHBEFAG *gene cluster expression. Corresponding DNA microarray studies with the *atpDCIHBEFAG *genes that encode a bacterial-like F_0_F_1 _complex revealed that only two of the nine genes (*atpD *and *atpC*) were expressed significantly higher in acetate by 3.2 and 1.8 fold, respectively: the remaining genes were either not detected or did not exhibit changes. Lastly, relative to central pathway genes for acetate and methanol utilization, it was unresolved how the *aha *and *atp *gene sets are expressed since the microarray data did not address this.

From the RT-PCT transcript abundance studies, three representative *aha *genes representing the archaeal-type A_0_A_1 _ATP synthase genes were highly expressed relative to the *atp *reporter genes (Figure 5C). Acetate cell growth conditions resulted in two-fold higher *aha *transcript levels relative to methanol cell growth. These genes were the most highly expressed in the cell regardless of the growth condition. In contrast, the bacterial-type F_0_F_1 _*atpD, atpA *and *atpG *genes were expressed at less than 2% of the level seen for the *ahaI, ahaC *and *ahaB *genes: this suggests a minor role for the *atp *genes in methanogenesis in contrast to the *aha *gene cluster.

### Acetate-induced genes

**Figure 6:**
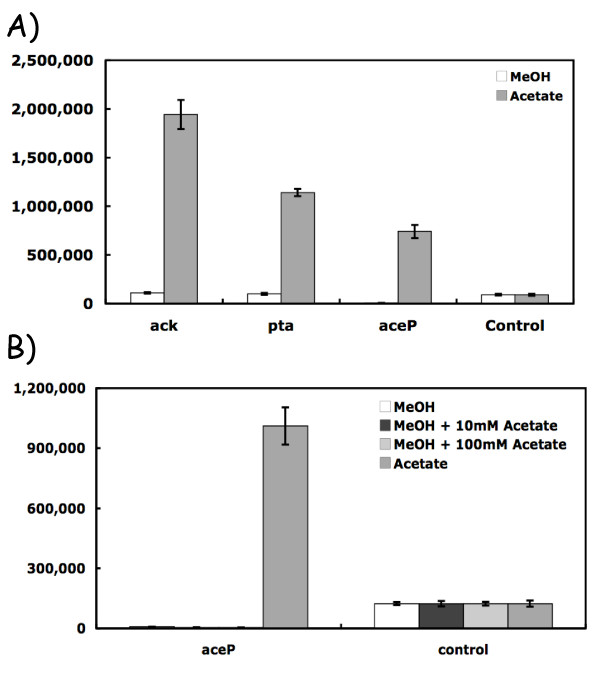
**Differential expression of genes induced in presence of acetate**. Panel A) The indicated genes include *ack *(acetate kinase), *pta *(phosphoacetyl transferase), and a gene designated *aceP *encoding a putative acetate uptake system. The RT-PCR data were determined as described in Materials. Panel B) Transcript abundance for *aceP *from cells grown in the presence or absence of the methanogenic substrate, methanol with the indicated amounts of acetate present.

One *M. acetivorans *gene of unknown function (MA4008) was revealed by our prior DNA microarray studies to be more highly expressed during acetate growth conditions relative to methanol cell growth (Lars Rohlin, personal communication). Inspection of the amino acid sequence revealed six trans-membrane spanning regions reminiscent of a membrane solute uptake system (Additional file 1, Figure S1 and discussed below). To extend these MA4008 gene expression findings, quantitative PCR experiments were performed (Methods, Figure 6A). MA4008 was expressed at a 125-fold higher level during acetate versus methanol cell growth conditions. Interestingly, when methanol was also present in the culture medium in addition to acetate, MA4008 expression was suppressed to a level seen when only methanol was present (ca. by 215 fold). This indicates that the MA4008 gene is expressed only when the energetically superior carbon substrate is absent, consistent with a proposed role in acetate uptake. The *M. acetivorans *MA4008 orf is designated *aceP *for its role in an acetate-dependent membrane function. Two other genes required for acetate utilization are *ack *(MA3606) and *pta *(MA3607) that encode acetate kinase and phosphoacetyl transferase, respectively ([15] Table 1). Quantitative PCR experiments (Figure 6A) established that both genes were highly expressed and at levels similar to *aceP *when acetate was the sole substrate. The 11-18-fold differential *pta *and *ack *gene expression findings are similar to previous reports in *M. acetivorans *and *M. thermophila *[6,16].

### Location of the *fpoP, hdrE, hdrA1, mrpA, pta, aceP*, and *ahaA *promoters

**Figure 7:**
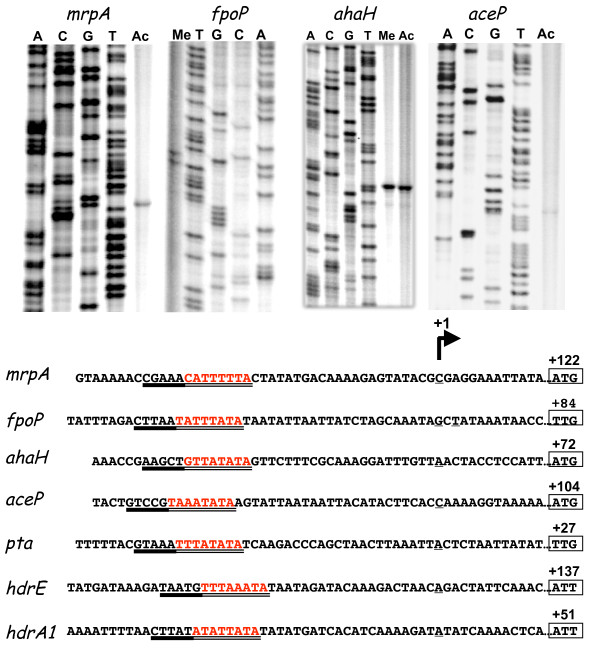
**Location of the mRNA 5'ends for the *hdrE1, hdrA1*, *mrpA, fpoP, pta, aceP*, and *ahaA *genes**. Top panel; Sequence gels for the *mrpA*, *fpoP, ahaA *and *aceP *genes along with the corresponding DNA ladders. RNA prepared from methanol or from acetate-grown cells is indicated by Me and Ac, respectively. Bottom panel: the alignment of the upstream DNA sequences relative to the start of transcription (+1 position). The position of the initiation codon is boxed where the numbering is relative to the start of transcription. The putative TATA-box sequences are double underlined and the BRE-regions are indicated by a solid underline. The mRNA 5' end positions for the *pta, hdrA1*, and *hdrE1 *genes were determined with a ubiquitous ladder (data not shown).

The mRNA 5' ends of the *fpoPABCDHIJJKLMNO, hdrED1, hdrA1-pfd*, *mrpABCDEFG, pta ack, aceP *and *ahaHIKECFABD *genes/clusters were determined to locate their corresponding promoter elements. Using primer extension methods (Figure 7A), all but one of the promoter elements were demonstrated to have long un-translated regions (UTR's) that range from 51 to 137 nucleotides in length. For example, the *aceP *5' mRNA end is located 104 nucleotides upstream of the translational start site. Similar findings were seen for the *mrpA, fpoP, ahaH, hdrE*, and *hdrA *genes. Only the *pta *gene had a relatively short UTR (i.e., 27 nt). We did not detect mRNA 5' ends for either *rnfX *or *hdrC1*. Alignment of all the upstream regions of these promoter elements (Figure 7A) revealed the highly conserved sequence present in other archaeal promoters, the TATA box (Figure 7B) located approximately 20-30 nt upstream of the +1 mRNA start site (discussed below). This site is bound by the TBP protein that aids RNA polymerase binding [17]. In contrast, the BRE box elements were not well conserved. When the UTR elements and the upstream regions were further examined using a suite of bioinformatics tools (Materials), no clearly discernable DNA sequence elements with either dyad symmetry or direct repeats were found. Similarly, no conserved regions within the RNA UTR's were seen for the coordinately expressed *hdrA1pfd *and *hdrC1B1 *genes sets.

## Discussion

Prior microarray and proteomic experiments reported transcript/protein ratios for a subset of the *M. acetivorans *genes addressed in this study [6,18]. However, by the limitations of the methods used, these studies did not provide expression ratios for many other key methanogenic pathway genes nor did they report information for other genes with potential roles in cell energy generation. Therefore quantitative PCR gene expression studies were undertaken here using *M. acetivorans *as a model to organism to examine which of the seemingly redundant gene copies in *Methanosarcina *species are utilized during growth on the alternative methanogenic substrates, acetate and methanol. As a result, we may interpret the resulting data as a readout of cell commitment to make RNA. From these experiments six points are readily apparent.

First, this study establishes the simultaneously high levels of gene expression for both a molybdenum-type (*fmdE1F1A1C1D1B1*) and a tungsten-type (*fwdD1B1A1C1*) formyl methanofuran dehydrogenase enzyme in *M. acetivorans *(Figure 1). In contrast, the *fmd2 *and *fwd2 *gene clusters were not. The co-expression of the *fmd1 *and *fwd2 *gene clusters during routine cell culture suggest that both tungsten and molybdate oxyanions are limiting during cell growth. Alternatively, the cell may somehow require the two gene sets to catalyze different reactions in methanogenic metabolism. Studies of the *Methanobacterium wolfei *and *Methanobacterium thermoautotrophicum *enzymes indicate that a tungsten-containing isoenzyme was constitutively expressed and that a molydate-containing isoenzyme was induced by molybdate ions [19]. Studies are in progress to establish if one or both of these oxyanion-metals modulate expression of the *M. acetivorans fwd1 *and/or *fmd1 *gene clusters. The *M. acetivorans *expression findings predict that the homologous *fmd1 *and the *fwd1-*type gene clusters in *M. barkeri *and in *M. mazei *are used to make the major formyl methanofuran dehydrogenase enzymes (Table 1). Interestingly, the *M. barkeri *genome lacks the annotated *fwd1 *tungsten-type enzyme.

Second, all sequenced *Methanosarcina *genomes contain multiple *hdr *genes encoding a membrane-type as well as a soluble-type heterodisulfide reductase (Table 1, Figure 2). Based on the transcript abundance studies in *M. acetivorans*, the membrane-type Hdr complex encoded by the *hdrED1 *genes was the most abundantly expressed gene cluster (Figure 2). This is consistent with the biochemical role for the membrane bound enzyme in *M. barkeri *[7]. However, given the high transcript levels for the *hdrA1 *and *hdrB1 *genes in cells grown with either acetate or methanol, a physiological role is hereby predicted for a soluble-type HdrABC heterodisulfide reductase in *M. acetivorans *metabolism, and by inference, in *M. mazei *and *M barkeri*. The presence of a poly-ferredoxin-like gene immediately downstream of the *hdrA1 *gene (Figure 2B) provides one candidate for electron transfer from primary electron donors (i.e., from methanol via either formyl methanofuran dehydrogenase, or from acetate via carbon monoxide dehydrogenase) to this Hdr soluble-type enzyme (discussed below). Transcript abundance for both the *hdrED1 *and *hdrA1B1 *genes were within the same magnitude observed for the *fpoN *and *fpoL *genes (Figure 3C) that encode subunits of the F420 H_2 _dehydrogenase needed for central carbon flow to carbon dioxide. Since genes for both a membrane-type and a soluble-type Hdr enzyme are co-expressed, this suggests that multiple pathways exist for electron transfer and/or energy conservation in *M. acetivorans*. By inference, the homologous *hdrA pfd *and *hdrC1B1*gene sets in *M. barkeri *and *M. mazei *are also highly expressed and operative. The energetic implication for having distinct Hdr-type enzymes is unknown. Possibilities include adaptation to different substrate levels and/or alternative modes of energy conservation [20].

Third, regarding the *M. acetivorans *sets of *frh, vhtG1*, and *vhtG2 *genes (Figure 3), plus the two electron transfer complexes encoded by *rnfXCDGEABY *and *mrpABCDEFG *genes (Figure 4), only the *vhtG1*, *rnf *and *mrp *gene sets were abundantly expressed. The *vhtG1A1C1D1*gene cluster encoding a methanophenazine-linked type hydrogenase was expressed at four- to six-fold higher levels during methanol growth conditions, and within the range seen for the *fpoL *and *fpoN *genes needed for methyl group oxidation for methanol and acetate metabolism. This is also in the range seen for methanol-dependent *fmdA1*, and *fwdA1 *expression (Figure 1). In contrast, no *vht *gene expression was detected in *M. acetivorans *when a *vht-uidA *promoter assay system was used [21]. Whether the high *vhtG1 *and *vhtC1 *mRNA levels detected here (Figure 3) versus the low values by the *vht-uidA *promoter assay is due to strain differences, cell growth, and/or in the analytical methods used is unknown. The *vhtG1A1C1 *like-hydrogenase genes are conserved among the three *Methanosarcina *strains (Table 1) where a more complex gene expression pattern is evident across these species [8,22]. Interestingly, the *M. acetivorans vht *mRNA expression pattern was similar to that seen in *M. mazei *[22], and a physiological role is implied for the *M. acetivorans vht *genes.

The *rnf *and *mrp *gene clusters are unique to the metabolism of *M. acetivorans *since related gene clusters are absent in either of the *M. mazei *and *M. barkeri *genomes (Table 1, [5,23]). As noted by Li, the *rnfXCDGEABY *gene products are logical candidates to fulfill the role of the Ech-type hydrogenases present in *M. mazei *and *M. barkeri *[10]. By this scheme, the Rnf complex would accept electrons derived from the carbon monoxide dehydrogenase (CODH) complex via an associated ferredoxin encoded by the complex. The membrane associated Rnf-type complex is then proposed to transfer electrons on to the membrane associated methanophenazine cofactor (MPH) that in turn is reoxidized by a membrane-type heterodisulfide reductase (e.g. HdrED). From the *hdr *transcript studies (Figure 2), this enzyme would be encoded by the *hdrED1 *gene set since *hdrD2 *expression was low. By an alternative model, one might envision a role for the Rnf complex in transferring electrons to the soluble heterodisulfide reductase complex encoded by the *hdrA1 pfd *and *hdrC1B1 *genes via protein-protein interactions. The poly-ferredoxin encoded by *pfd *(MA2867) from the soluble-type heterodisulfide gene cluster is one candidate to interact with one of the unique Rnf complex proteins such as RnfX or RnfY. Either model is compatible with the essentiality for Rnf based on the effect of an *rnf *deletion strain that is unable to grow with acetate as a sole carbon supply. Little biochemical data exist to distinguish among these possibilities.

**Figure 8:**
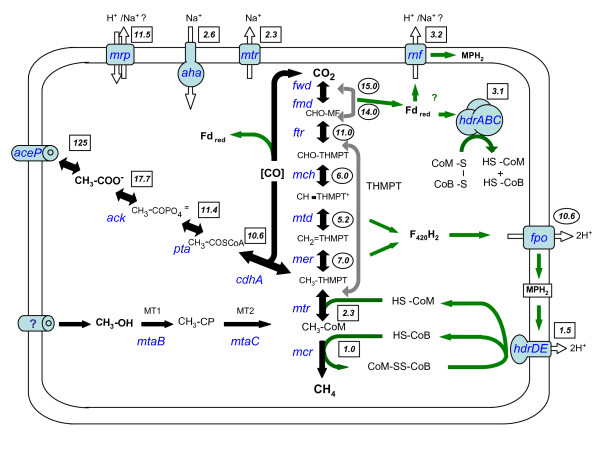
**Overview of differential gene expression in *M. acetivorans *in response to methanol versus acetate utilization**. A boxed number indicates the fold-increase in mRNA levels seen for the indicated gene(s) during acetate versus methanol growth conditions. A circled number indicates the fold-increase in mRNA levels during methanol versus acetate growth conditions. All data are from this study except for the *mcr, mtr, mer, mtd, mch*, and *ftr *gene ratio data derived from a prior microarray study [6]. The genes/enzymes are: *ack*, acetate kinase; *pta*, phosphotransacetylase; *cdh*, carbon monoxide dehydrogenase; MT1, *mtaB2 *methyl transferase 1; MT2, *mtaA*, methyltransferase 2; *mcr*, methylcoenzyme M reductase; *mtr*, methyl -H4 MPT:HSCoM methyltransferase; *mer*, methylene -H4 MPT reductase; *hmd*, methylene -H4 MPT dehydrogenase; *mch*, methenyl -H4 MPT cyclohydrolyase; *ftr*, formyl MFR:H4MPT formyl transferase; *fmd*, formyl methanofuran dehydrogenase Mo-type; *fwd*, formyl methanofuran dehydrogenase W-type; *fpo*, F420 H2 dehydrogenase; *hdr*, heterodisulfide reductase; *rnf*, Rnf-type complex; *mrp*, Mrp-type complex. The control gene was MA3998. Methanophenazine is represented by MPH. The proposed acetate transporter protein is indicated by AceP while the unknown transporter(s) for one carbon compounds is indicated by a question mark.

Based on the role of the Mrp complex in cytoplasmic pH homeostasis in *Bacillus halodurans*, a similar function was proposed for the *M. acetivorans *Mrp-like complex [10]. Both belong to the Group I class of proteins and exhibit similar gene compositions and gene order [24]. Interestingly, several alternative roles have been suggested for the bacterial Mrp genes and include exchange of another type of mono-valent ion, in detoxification, and in interactions with another cellular enzyme to form a membrane complex somehow associated with cellular ion partitioning [24]. A role for the *M. acetivorans *gene products in cytoplasmic pH homeostasis or the other above roles would make it distinct from other *Methanosarcina *species since related *mrp *genes are absent in the other sequenced genomes (Table 1). In this regard, phenotypic analysis of *M. acetivorans mrp *mutants will be of special interest. The high similarity of the *M. acetivorans mrp *genes relative to those in the bacteria, suggest an origin in the methanogen by lateral gene transfer event from a Group I organism. Do the *M. acetivorans mrp *and *rnf *transcript abundance data provide additional clues about the roles of either Mrp or Rnf? The genes for both are among the most highly expressed in the cell (Figure 4), where the *mrp *gene expression pattern is similar to levels for the *ack *and *pta *genes needed for acetate utilization (Figure 6). The 8-fold higher *mrp *expression level relative to methanol growth approximates the 8-12 fold seen for the *ack *and *pta *genes (Figure 8) in support of a primary role in acetate-dependent metabolism, rather than in detoxification and/or ion homeostasis. In contrast, a second pattern of gene expression is seen for the central pathway genes involved in one carbon oxidations (*mer, mtd, mch*, fpo, and *ftr*) that are all more highly expressed by 5 to 11 fold when methanol is the sole substrate (Figure 8). A third set of genes required for both acetate and methanol metabolism are differentially expressed at an intermediate level (e.g., *mtr *genes, 2.3-fold; *hdrDE*, 1.2-fold, and *hdrABC*, 3-fold). The *rnf *gene expression pattern (i.e., 2.4 fold higher level with acetate) falls in this group. It is interesting to speculate that some of these genes may be controlled in response to electron flow rather than the carbon supply (e.g., acetate versus methanol availability).

Forth, the quantitative ATPase gene expression studies demonstrate that the archaeal-type A_0_A_1 _ATP synthase encoded by the *ahaHIKECFABD *genes are among the most highly expressed genes in the cell (Figure 5). In contrast, transcript abundance for the bacteria type *atpDCIHBEFAG *genes was about 175-fold lower than these *aha *cluster genes during either acetate or methanol growth. Although it is conceivable that the *atp*-type genes may be significantly expressed under unknown growth conditions, an alternative possibility is that they constitute a "dead-ended" lateral gene transfer event [23]. Interestingly, the deletion of the *atp *gene region of *M. acetivorans *conferred no phenotype [25]. The *atpX *gene present in the *M. acetivorans *and *M. barkeri *genomes is conserved in some, but not all bacterial-like ATP synthase operons. It is present in the *Rhodoferax ferrireducens *DSM 15236, *Desulfuromonas acetoxidans *DSM 684 and *Shewanella frigidimarina *NCIMB genomes (gene alignments not shown). Since the synteny of *atpX *in the above operons is conserved, *atpX *is not due to an isolated insertion event in the *M. acetivorans *genome.

**Figure 9:**
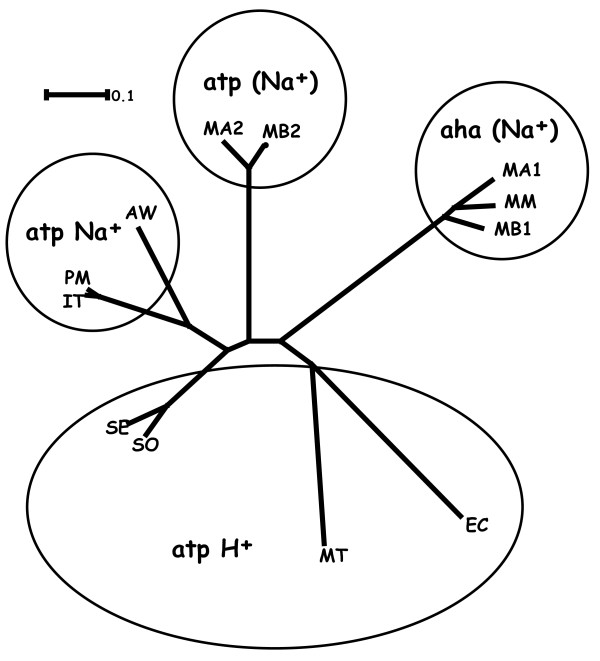
**Phylogenic tree of the *atp *and *aha *ATP synthase proteolipid subunit c for the methanogens *M. acetivorans, M. mazei*, and *M. barkeri*, and for the bacterial homologs indicated in reference **[26]. The predicted or experimentally determined ion transferred is indicated from data provided in Additional file 2, Figure S2.

Biochemical studies have identified essential amino acids involved in translocation of sodium ions by the proteolipid c subunit of the *Ilyobacter tartaricus *ATPase [26]. To address whether Na^+ ^or H^+ ^ions are transported by the *M. acetivorans *archaeal-type A_0_A_1 _ATP synthase, the *ahaK *gene encoding proteolipid *c *subunit was aligned with the corresponding subunits of *I. tartaricus *plus other well studied microorganisms (Additional file 2, Figure S2). Four amino acid residues at positions 32, 63, 65, and 66 in the *I. tartaricus *protein to specify Na^+ ^ion movement [26]. These four residues are conserved in *M. acetivorans*, in contrast to *E. coli *that is a proton translocating enzyme. This suggests the archaeal-type A_0_A_1 _ATP synthase also transfers Na^+ ^ions rather than protons to form ATP, in keeping with the example of *Pyrococcus furiosus *[27]. Furthermore, the archaeal type *ahaK *subunit in the three *Methanosarcina *strains form a distinct protein subclass given the presence of an additional three amino acids relative to position 14 of the *I. tartaricus *subunit, and a three amino acid deletion corresponding to position 47-49 of *I. tartaricus*. Amino acid alignments of the A_0_A_1 _ATP synthases subunits from the *M. mazei *and *M. barkeri *proteolipids suggest the same conclusion for these highly related archaeal complexes (Additional file 2, Figure S2). Interestingly, the alignment of the c proteolipid subunit (*atpE*) of the *M. acetivorans *bacterial-type F_0_F_1 _synthase also suggests specificity for Na^+ ^ions. A neighbor-joining tree of the archaeal and bacterial c-type polypeptides (Figure 9) reveals a relatively conserved origin of the archaeal-type A_0_A_1 _ATP synthase in the *Methanosarcina *species. Strikingly, the bacterial-type F_0_F_1 _synthase genes present in *M. acetivorans *and *M. barkeri *are more distantly related to either the archaeal or bacterial type enzymes. This branch of ATP metabolism genes/proteins remains poorly understood and awaits further study.

Fifth, a candidate gene encoding a potential acetate uptake system for *M. acetivorans *was identified (Figure 6). This gene exhibits the same expression patterns as the *ack *and *pta *genes needed for activation of the methanogenic substrate following its entry into the cell. Expression of *aceP *was suppressed by the energetically favorable substrate, methanol (Figure 6B). The AceP protein is predicted to have six transmembrane-spanning alpha-helical regions (Additional file 1, Figure S1). Noteworthy, *aceP *homologs are present in other methanogens including *M. mazei, M. barkeri, M. maripaludis*, and *M. hungatei*, and they constitute a distinct class of archaea transporters. Related genes are also present in many bacterial species (Additional file 3, Figure S3), suggesting the possibility of a lateral gene transfer event from a bacterium into the *Methanosarcina *sp. as was proposed as one explanation for their large genome sizes [23]. Experiments are in progress to characterize the membrane function of the *M. acetivorans *protein since no archaeal or bacterial homologs shown in Additional file 3, Figure S3 have been examined to date.

### Carbon control in the Archaea

Considerable information is available concerning carbon control of gene expression in bacterial and eukaryal systems, but little is yet known about related carbon control in the Archaea. Few studies have been reported for any archaeal species but include microarray studies in *Pyrococcus furiosus *[28], *M. mazei *[29,30], and *M. acetivorans *[6]. The present experiments extend these studies to address a larger set of genes needed for carbon flow and electron transfer leading to methane formation from two key methanogenic substrates (Figure 8). It provides a foundation of RNA transcript abundance and 5' end data to begin exploring regulatory controls in this organism at the level of regulated mRNA synthesis and turnover. Little is known about the relative contributions of archaea transcription factors, translation factors, and/or small RNA's in gene regulation in the *Methanosarcina *species to provide the distinct patterns of gene expression observed here. *M. acetivorans *clearly maintains a cellular commitment to dynamically control transcript levels in response to methanogenic substrate type where two major gene families are further defined by this study.

## Conclusion

Of the twenty *M. acetivorans *gene clusters examined in this study, all but four were differentially expressed by 2 to 200-fold during acetate versus methanol cell growth (Figures 1, 2, 3, 4, 5, 6). The majority of these queried genes are present all sequenced *Methanosarcina *genomes that include *M. acetivorans, M. mazei *and *M. barkeri *(Table 1) and include the genes for multiple heterodisulfide reductase and hydrogenase-like enzymes. Exceptions are the *echABCDEF, vhoGAC, rnfXCDGEABY*, and *mrpABCDEFG *genes that encode known or predicted electron transfer complexes for ion movement and/or electron transfer. A number of the above orfs are assigned new gene designations to aid in their description. The *M. acetivorans *gene expression data (Figures 1, 2, 3, 4, 5, 6, 7, 8) provides a foundation to understand how energy-yielding pathways are regulated in this model organism and in related methanogens. It is unknown if this control occurs by the actions of classical transcription factors like those found in bacteria and eukaryotes, and/or by RNA control mechanisms involving attenuation, regulated termination and/or small RNAs.

## Methods

### Cell culture

*Methanosarcina acetivorans *C2A [1] was cultivated in a mineral medium that contained (in grams per liter): NaCl, 11.69 g; MgSO_4 _7H_2_O, 12.32 g; KCl, 0.76 g; CaCl_2_·2H_2_O, 0.14 g; NH_4_Cl, 0.5 g; Resazurin solution (10,000 × stock solution), 0.1 ml; trace metal solution (100×) 10 ml [29]; vitamin solution (100×) 10 ml [29]; HCl (12.1 N) 0.5 ml; Na_2_HPO_4 _7H_2_O, 1.12 g; cysteine-HCl H_2_O, 0.25 g; Na_2_CO_3_, 3.0 g. An atmosphere (80:20) of nitrogen to carbon dioxide was used in the vessel headspace. Following sterilization, the medium was supplemented with filter-sterilized 0.1 ml 50% methanol or 0.2 ml 5 M acetate per 10 ml medium as previously described [30].

### RNA purification

For RNA isolation, cultures of *M. acetivorans *C2A cells were grown on acetate or methanol with serial transfer of three times to mid-exponential phase before cell harvest. Total RNA was purified from 10 ml of cell samples using the RNAwiz (Ambion Austin, TX) following the manufacturer's instructions. The purified RNA was treated with DNase I as described [31,32].

### Quantitative RT-PCR

The real time reverse transcription (RT-PCR) reactions were performed using Superscript II reverse transcriptase (Invitrogen Carlsbad, CA) according to the manufacturers recommended protocol using random primers and 1 μg of total RNA. A mock reaction without Superscript was run to evaluate for the presence of genomic DNA contamination. To remove complementary RNA, 1 μl RNase H was added to mixture and incubated for 20 min at 37°C. The RNase was then heat inactivated at 70°C for 15 min. The cDNA from the RT reaction was diluted 10 fold, and 1 μl of the diluted cDNA was subsequently used in a 30 μl iQ SYBR green supermix according to the manufactures recommendations following addition of 1.5 μl DMSO. The real time PCR reactions were conducted on a Biorad iCycler (Biorad, Hercules, CA) or an Eppendorf Realtime^2 ^(Eppendorf, Westbury, NY) using a four-step program consisting of, denaturing, annealing, extension, and acquisition steps. The RT-PCR primers were created by a modified version of MyPROBES [32]. The PCR product lengths were in a range of 100-200 bp, the melting temperature was in the range of 55-66°C, the GC content was 55-65%, and the primer length was 17-22 bases (Additional file 4, Table S1). The primers were tested against serial dilution of genomic DNA (10^6 ^to 10^2 ^copies) to generate a standard curve for each gene tested. The products were also visualized on agarose gels to establish the generation of a unique product of the correct size for each gene probe. Each gene expression value was then determined in triplicate for each of the three biological samples in conjunction with a genomic DNA serial dilution standard. Melting curves were analyzed to establish that non-specific amplification had not occurred (i.e., biphasic vs mono-phasic for a single product). The reported copy number was calculated from a total of nine data points. Each gene was also tested against the mock reaction. The gene expression data for each gene was compared to a reference gene (MA3998) that showed no significant up or down regulation in microarray experiments of Li, et al. [6]. In an independent approach, all qPCR signals were also normalized to the total amount of RNA used in the experiment, and in a separate analysis, to the RNA for the *mcr *genes (MA4546-4550) that encode methyl coenzyme M reductase. The results from the latter two approaches were in excellent agreement to the MA3998 normalization procedure. Values are reported in transcript copy number per 5 μg total RNA.

### Primer extension analysis

To determine mRNA 5' ends, primer extension reactions were performed as described previously [33] using gene specific primers which were located approximately 60 bases downstream of the ATG start codons of the *mrpA, hdrE, hdrA, aceP, ahaH, pta*, and *fpoP *genes (see Additional file 4, Table S1 listing each primer). Total RNA was isolated described above. A total of 30 μg of RNA was used in each primer extension reaction: the primer and RNA was heated to 85°C for 10 min, and then slowly cooled to 45°C: ^33^P-labeled dATP and unlabeled dCTP, dGTP, and dTTP were added to the mixture, and reverse transcription was then performed at 50°C using Superscript III Reverse Transcriptase (Invitrogen Carlsbad, CA) according to manufactures recommendations. The reaction was stopped by sequentially adding 5 μl 3 M sodium acetate (pH 5.2) and 150 μl 100% ice-cold ethanol followed by overnight incubation at -20°C. The cDNA's was precipitated at 13,000 rpm at 4°C for 35 min. For generation of fragments of the indicated regulatory region was cloned into TOPO-PCR4 vector (Invitrogen Carlsbad, CA). The Sequtherm Excel II Kit (Epicentre Madison, WI) was used to perform sequencing reactions of the DNA regions cloned into TOPO-PCR4 using the above primers to confirm the intended sequences. The extension and sequencing products were resolved on a 6.0% sequencing gel and exposed to a phosphorimager screen as previously described [32].

### Informatics analysis and data visualization

Protein similarities were determined using BLAST [34], the alignment and the phylogentic tree of proteins were done with clustalw [35] and the visualization of the trees were done with splitTree4 [36]. Upstream DNA regions were searched for palindromic and repeated motifs using simple Perl script software written in house. Similar searches were also performed for conserved elements in the UTR regions.

## Authors' contributions

LR performed the gene expression and informatics analysis of the genomes, and contributed to the concepts and strategy for performing the study. RG provided critical comments to improve the experimental design, and manuscript layout. All authors were involved in analyzing all the data, read and approved the final manuscript.

## Supplementary Material

Additional file 1 **Figure S1. Amino acid alignment of the acetate induced membrane protein from *M. acetivorans *and several other organisms**. *Bacillus Anthracis str. Ames, Burkholderia xenovorans, Haemophilus somnus, Pasteurella multocida, MeOHP Methanococcoides burtonii, UnkP Methanosarcina mazei, UnkP Methanosarcina acetivorans, MeOHP Methanosarcina acetivorans, MeOHP Methanosarcina barkeri, MeOHP Methanosarcina mazei, Methanothermobacter thermautotrophicus, Desulfovibrio desulfuricans, Chromobacterium violaceum, Shewanella oneidensis MR-1, Dehalococcoides sp. CBDB1, Erwinia carotovora, Photorhabdus luminescens, Yersinia pestis KIM, Salmonella typhimurium, Escherichia coli, Geobacter metallireducens, Pelobacter carbinolicus, AceP Methanosarcina acetivorans, AceP Methanosarcina barkeri, AceP Methanosarcina mazei, Methanospirillum hungateii, Anaeromyxobacter dehalogenans, AceP Methanococcoides burtonii, Methanococcus maripaludis, Sulfolobus acidocaldarius, Sulfolobus solfataricus P2, Picrophilus torridu, Thermoplasma acidophilum, Thermoplasma volcanium GSS1*. Click here for file

Additional file 2 **Figure S2. Amino acid alignment of the proteolipid c subunits of the ATP synthases from *M. acetivorans *and several other organisms**. The bacterial-type (MA2436, MA2) and the archaeal-type gene cluster/protein (MA4154, MA1) from *M. acetivorans *are shown with the corresponding sequences for *Ilyobacter tartaricus (IT), Acetobacterium woodii (AW), Propionigenium modestum (PM), M. barkeri (MB), E. coli (EC), M. tuberculosis (MT), Spinachia oleracea (SO), and Synechococcus elongatus (SE)*. Numbering is relative to the start of translation of *Ilyobacter tartaricus *[26]. Amino acids are indicated by color: orange (GPST), red (HKR), blue (FWY, green (ILMV). Click here for file

Additional file 3 **Figure S3. Phylogenic tree of the pudative *aceP *membrane protein from *M. acetivorans***. *Bacillus Anthracis str. Ames, Burkholderia xenovorans, Haemophilus somnus, Pasteurella multocida, MeOHP Methanococcoides burtonii, UnkP Methanosarcina mazei, UnkP Methanosarcina acetivorans, MeOHP Methanosarcina acetivorans, MeOHP Methanosarcina barkeri, MeOHP Methanosarcina mazei, Methanothermobacter thermautotrophicus, Desulfovibrio desulfuricans, Chromobacterium violaceum, Shewanella oneidensis MR-1, Dehalococcoides sp. CBDB1, Erwinia carotovora, Photorhabdus luminescens, Yersinia pestis KIM, Salmonella typhimurium, Escherichia coli, Geobacter metallireducens, Pelobacter carbinolicus, AceP Methanosarcina acetivorans, AceP Methanosarcina barkeri, AceP Methanosarcina mazei, Methanospirillum hungateii, Anaeromyxobacter dehalogenans, AceP Methanococcoides burtonii, Methanococcus maripaludis, Sulfolobus acidocaldarius, Sulfolobus solfataricus P2, Picrophilus torridu, Thermoplasma acidophilum, Thermoplasma volcanium GSS1*.
Click here for file

Additional file 4 **Table S1. Oligonucleotides used in this study**. Description: This table provides the nucleotide sequence of all oligonucleotides used for PCR-based experiments.
Click here for file

## References

[B1] Sowers KR Baron SF Ferry JG *Methanosarcina acetivorans *sp. nov., an Acetotrophic Methane-Producing Bacterium Isolated from Marine Sediments Appl Environ Microbiol 1984 47 5 971 978 16346552 10.1128/aem.47.5.971-978.1984 PMC240030

[B2] Ferry JG (ed) Methanogenesis; Ecology, Physiology, Biochemistry and Genetics 1993 New York: Chapman and Hall

[B3] Deppenmeier U The unique biochemistry of methanogenesis Prog Nucleic Acid Res Mol Biol 2002 71 223 283 full_text 12102556 10.1016/s0079-6603(02)71045-3

[B4] Thauer RK Biochemistry of methanogenesis: a tribute to Marjory Stephenson Microbiology 1998 144 9 2377 2406 10.1099/00221287-144-9-2377 9782487

[B5] Galagan JE Nusbaum C Roy A Endrizzi MG Macdonald P FitzHugh W Calvo S Engels R Smirnov S Atnoor D The genome of *Methanosarcina acetivorans *reveals extensive metabolic and physiological diversity Genome Res 2002 12 4 532 542 10.1101/gr.223902 11932238 PMC187521

[B6] Li L Li Q Rohlin L Kim U Salmon K Rejtar T Gunsalus RP Karger BL Ferry JG Quantitative proteomic and microarray analysis of the archaeon *Methanosarcina acetivorans *grown with acetate versus methanol J Proteome Res 2007 6 2 759 771 10.1021/pr060383l 17269732 PMC2577390

[B7] Kunkel A Vaupel M Heim S Thauer RK Hedderich R Heterodisulfide reductase from methanol-grown cells of *Methanosarcina barkeri *is not a flavoenzyme Eur J Biochem 1997 244 1 226 234 10.1111/j.1432-1033.1997.00226.x 9063468

[B8] Guss AM Mukhopadhyay B Zhang JK Metcalf WW Genetic analysis of mch mutants in two *Methanosarcina *species demonstrates multiple roles for the methanopterin-dependent C-1 oxidation/reduction pathway and differences in H(2) metabolism between closely related species Mol Microbiol 2005 55 6 1671 1680 10.1111/j.1365-2958.2005.04514.x 15752192

[B9] Nelson MJ Ferry JG Carbon monoxide-dependent methyl coenzyme M methylreductase in acetotrophic *Methosarcina *spp J Bacteriol 1984 160 2 526 532 6501214 10.1128/jb.160.2.526-532.1984 PMC214766

[B10] Li Q Li L Rejtar T Lessner DJ Karger BL Ferry JG Electron transport in the pathway of acetate conversion to methane in the marine archaeon *Methanosarcina acetivorans* J Bacteriol 2006 188 2 702 710 10.1128/JB.188.2.702-710.2006 16385060 PMC1347274

[B11] Blanco-Rivero A Leganes F Fernandez-Valiente E Calle P Fernandez-Pinas F mrpA, a gene with roles in resistance to Na+ and adaptation to alkaline pH in the cyanobacterium *Anabaena *sp. PCC7120 Microbiology 2005 151 Pt 5 1671 1682 10.1099/mic.0.27848-0 15870474

[B12] Sun H Shi W Genetic studies of mrp, a locus essential for cellular aggregation and sporulation of *Myxococcus xanthus* J Bacteriol 2001 183 16 4786 4795 10.1128/JB.183.16.4786-4795.2001 11466282 PMC99533

[B13] Ito M Guffanti AA Oudega B Krulwich TA mrp, a multigene, multifunctional locus in *Bacillus subtilis *with roles in resistance to cholate and to Na+ and in pH homeostasis J Bacteriol 1999 181 8 2394 2402 10198001 10.1128/jb.181.8.2394-2402.1999 PMC93663

[B14] Dzioba-Winogrodzki J Winogrodzki O Krulwich TA Boin MA Hase CC Dibrov P The *Vibrio cholerae *Mrp system: cation/proton antiport properties and enhancement of bile salt resistance in a heterologous host J Mol Microbiol Biotechnol 2009 16 3-4 176 186 10.1159/000119547 18311075 PMC2640445

[B15] Latimer MT Ferry JG Cloning, sequence analysis, and hyperexpression of the genes encoding phosphotransacetylase and acetate kinase from *Methanosarcina thermophila* J Bacteriol 1993 175 21 6822 6829 8226623 10.1128/jb.175.21.6822-6829.1993 PMC206805

[B16] Singh-Wissmann K Ferry JG Transcriptional regulation of the phosphotransacetylase-encoding and acetate kinase-encoding genes (pta and ack) from *Methanosarcina thermophila* J Bacteriol 1995 177 7 1699 1702 7896690 10.1128/jb.177.7.1699-1702.1995 PMC176795

[B17] Bell SD Kosa PL Sigler PB Jackson SP Orientation of the transcription preinitiation complex in archaea Proc Natl Acad Sci USA 1999 96 24 13662 13667 10.1073/pnas.96.24.13662 10570129 PMC24121

[B18] Li Q Li L Rejtar T Karger BL Ferry JG Proteome of *Methanosarcina acetivorans *Part I: an expanded view of the biology of the cell J Proteome Res 2005 4 1 112 128 10.1021/pr049832c 15707366

[B19] Hochheimer A Hedderich R Thauer RK The formylmethanofuran dehydrogenase isoenzymes in *Methanobacterium wolfei *and *Methanobacterium thermoautotrophicum*: induction of the molybdenum isoenzyme by molybdate and constitutive synthesis of the tungsten isoenzyme Arch Microbiol 1998 170 5 389 393 10.1007/s002030050658 9818358

[B20] Thauer RK Kaster AK Seedorf H Buckel W Hedderich R Methanogenic archaea: ecologically relevant differences in energy conservation Nat Rev Microbiol 2008 6 8 579 591 10.1038/nrmicro1931 18587410

[B21] Guss AM Kulkarni G Metcalf WW Differences in hydrogenase gene expression between *Methanosarcina acetivoran*s and *Methanosarcina barkeri* J Bacteriol 2009 191 8 2826 2833 10.1128/JB.00563-08 19201801 PMC2668380

[B22] Deppenmeier U Blaut M Lentes S Herzberg C Gottschalk G Analysis of the vhoGAC and vhtGAC operons from *Methanosarcina mazei *strain Go1, both encoding a membrane-bound hydrogenase and a cytochrome b Eur J Biochem 1995 227 1-2 261 269 10.1111/j.1432-1033.1995.tb20383.x 7851393

[B23] Deppenmeier U Johann A Hartsch T Merkl R Schmitz RA Martinez-Arias R Henne A Wiezer A Bäumer S Jacobi C Brüggemann H Lienard T Christmann A Bömeke M Steckel S Bhattacharyya A Lykidis A Overbeek R Klenk HP Gunsalus RP Fritz HJ Gottschalk G The genome of *Methanosarcina mazei*: evidence for lateral gene transfer between bacteria and archaea J Mol Microbiol Biotechnol 2002 4 4 453 461 12125824

[B24] Swartz TH Ikewada S Ishikawa O Ito M Krulwich TA The Mrp system: a giant among monovalent cation/proton antiporters? Extremophiles 2005 9 5 345 354 10.1007/s00792-005-0451-6 15980940

[B25] Saum R Schlegel K Meyer B Muller V The F1FO ATP synthase genes in *Methanosarcina acetivorans *are dispensable for growth and ATP synthesis FEMS Microbiol Lett 2009 300 2 230 236 10.1111/j.1574-6968.2009.01785.x 19796137

[B26] Meier T Polzer P Diederichs K Welte W Dimroth P Structure of the rotor ring of F-Type Na+-ATPase from *Ilyobacter tartaricus* Science 2005 308 5722 659 662 10.1126/science.1111199 15860619

[B27] Pisa KY Huber H Thomm M Muller V A sodium ion-dependent A1AO ATP synthase from the hyperthermophilic archaeon *Pyrococcus furiosus* FEBS J 2007 274 15 3928 3938 10.1111/j.1742-4658.2007.05925.x 17614964

[B28] Schut GJ Brehm SD Datta S Adams MW Whole-genome DNA microarray analysis of a hyperthermophile and an archaeon: *Pyrococcus furiosus *grown on carbohydrates or peptides J Bacteriol 2003 185 13 3935 3947 10.1128/JB.185.13.3935-3947.2003 12813088 PMC161589

[B29] Wolfe RS Higgins IJ Quayle JR Microbial biochemistry of methane; a study in contrasts Microbial Biochemistry 1979 Baltimore: University Park Press 267 300

[B30] Sowers KR Robertson DE Noll D Gunsalus RP Roberts MF N^e^-acetyl-b-lysine: an osmolyte synthesized by methanogenic Archaebacteria Proc Natl Acad Sci USA 1990 87 23 9083 9087 10.1073/pnas.87.23.9083 2123548 PMC55108

[B31] Oh MK Rohlin L Kao KC Liao JC Global expression profiling of acetate-grown *Escherichia coli* J Biol Chem 2002 277 15 13175 13183 10.1074/jbc.M110809200 11815613

[B32] Rohlin L Trent JD Salmon K Kim U Gunsalus RP Liao JC Heat shock response of *Archaeoglobus fulgidus* J Bacteriol 2005 187 17 6046 6057 10.1128/JB.187.17.6046-6057.2005 16109946 PMC1196131

[B33] Sowers KR Thai TT Gunsalus RP Transcriptional regulation of the carbon monoxide dehydrogenase gene (*cdhA*) in *Methanosarcina thermophila* J Biol Chem 1993 268 31 23172 23178 7693685

[B34] Altschul SF Madden TL Schaffer AA Zhang J Zhang Z Miller W Lipman DJ Gapped BLAST and PSI-BLAST: a new generation of protein database search programs Nucleic Acids Res 1997 25 17 3389 3402 10.1093/nar/25.17.3389 9254694 PMC146917

[B35] Larkin MA Blackshields G Brown NP Chenna R McGettigan PA McWilliam H Valentin F Wallace IM Wilm A Lopez R Clustal W and Clustal X version 2.0 Bioinformatics 2007 23 21 2947 2948 10.1093/bioinformatics/btm404 17846036

[B36] Huson DH Bryant D Application of phylogenetic networks in evolutionary studies Mol Biol Evol 2006 23 2 254 267 10.1093/molbev/msj030 16221896

