# Supplementary figures and images for: Carbon-dependent control of electron transfer and central carbon pathway genes for methane biosynthesis in the Archaean, Methanosarcina acetivorans strain C2A
Source: BMC Microbiol. 2010 Feb 23;10:62. doi: 10.1186/1471-2180-10-62 (PMC2838876; doi:10.1186/1471-2180-10-62)

## Secondary structure

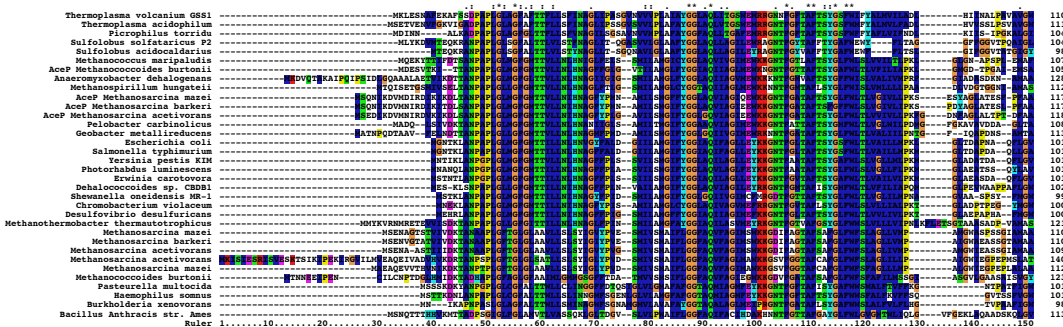

## Secondary structure

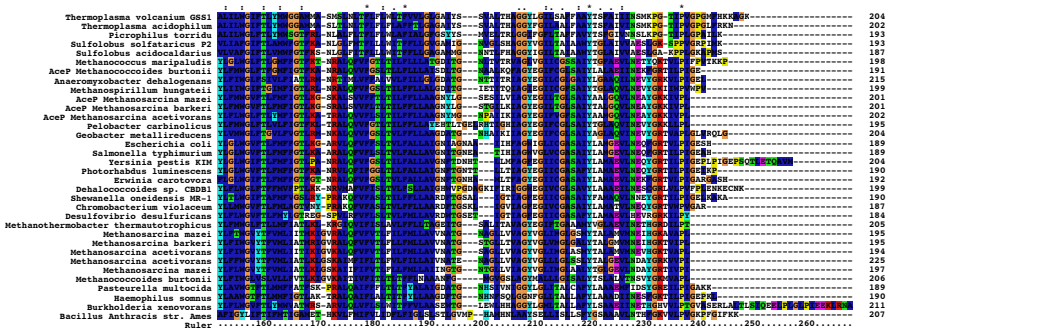

Supplement: Additional file 1 — Figure S1. Amino acid alignment of the acetate induced membrane protein from M. acetivorans and several other organisms. Bacillus Anthracis str. Ames, Burkholderia xenovorans, Haemophilus somnus, Pasteurella multocida, MeOHP Methanococcoides burtonii, UnkP Methanosarcina mazei, UnkP Methanosarcina acetivorans, MeOHP Methanosarcina acetivorans, MeOHP Methanosarcina barkeri, MeOHP Methanosarcina mazei, Methanothermobacter thermautotrophicus, Desulfovibrio desulfuricans, Chromobacterium violaceum, Shewanella oneidensis MR-1, Dehalococcoides sp. CBDB1, Erwinia carotovora, Photorhabdus luminescens, Yersinia pestis KIM, Salmonella typhimurium, Escherichia coli, Geobacter metallireducens, Pelobacter carbinolicus, AceP Methanosarcina acetivorans, AceP Methanosarcina barkeri, AceP Methanosarcina mazei, Methanospirillum hungateii, Anaeromyxobacter dehalogenans, AceP Methanococcoides burtonii, Methanococcus maripaludis, Sulfolobus acidocaldarius, Sulfolobus solfataricus P2, Picrophilus torridu, Thermoplasma acidophilum, Thermoplasma volcanium GSS1. [file 1471-2180-10-62-S1.PDF]

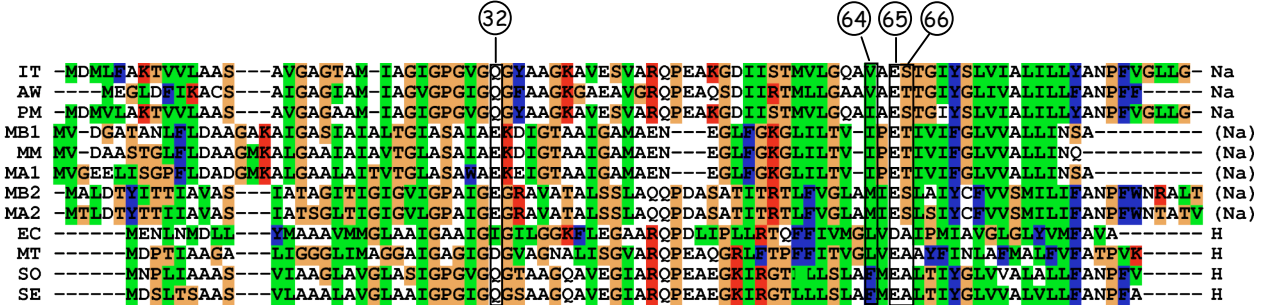

Supplement: Additional file 2 — Figure S2. Amino acid alignment of the proteolipid c subunits of the ATP synthases from M. acetivorans and several other organisms. The bacterial-type (MA2436, MA2) and the archaeal-type gene cluster/protein (MA4154, MA1) from M. acetivorans are shown with the corresponding sequences for Ilyobacter tartaricus (IT), Acetobacterium woodii (AW), Propionigenium modestum (PM), M. barkeri (MB), E. coli (EC), M. tuberculosis (MT), Spinachia oleracea (SO), and Synechococcus elongatus (SE). Numbering is relative to the start of translation of Ilyobacter tartaricus [26]. Amino acids are indicated by color: orange (GPST), red (HKR), blue (FWY, green (ILMV). [file 1471-2180-10-62-S2.PDF]

0.1

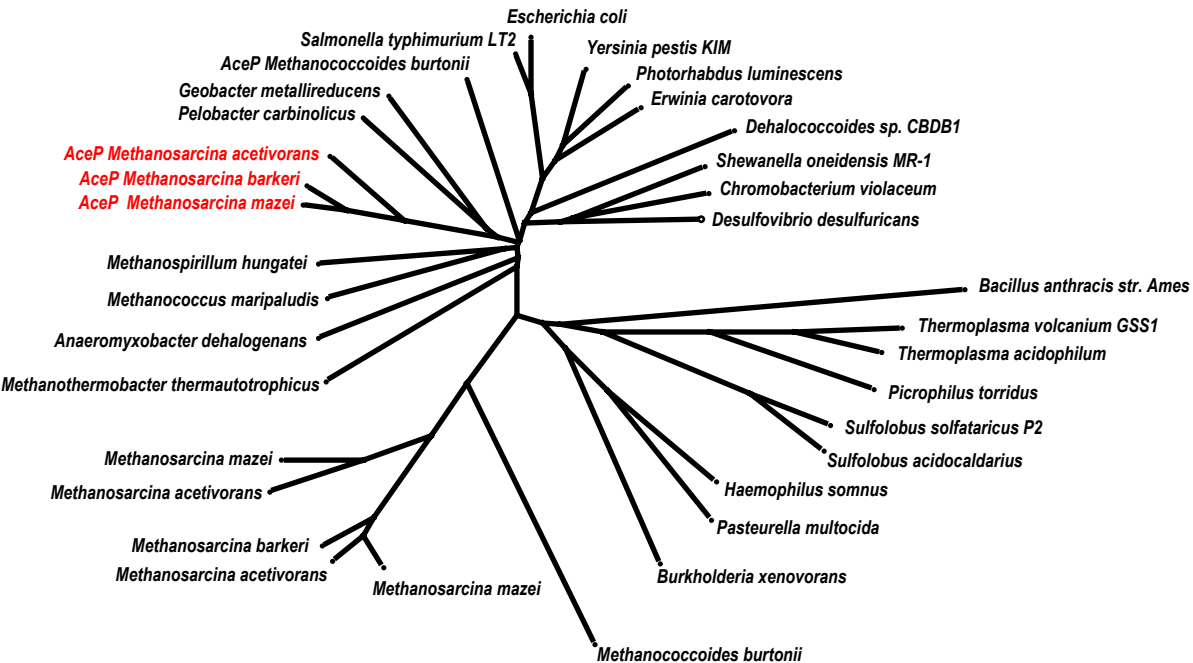

Supplement: Additional file 3 — Figure S3. Phylogenic tree of the pudative aceP membrane protein from M. acetivorans. Bacillus Anthracis str. Ames, Burkholderia xenovorans, Haemophilus somnus, Pasteurella multocida, MeOHP Methanococcoides burtonii, UnkP Methanosarcina mazei, UnkP Methanosarcina acetivorans, MeOHP Methanosarcina acetivorans, MeOHP Methanosarcina barkeri, MeOHP Methanosarcina mazei, Methanothermobacter thermautotrophicus, Desulfovibrio desulfuricans, Chromobacterium violaceum, Shewanella oneidensis MR-1, Dehalococcoides sp. CBDB1, Erwinia carotovora, Photorhabdus luminescens, Yersinia pestis KIM, Salmonella typhimurium, Escherichia coli, Geobacter metallireducens, Pelobacter carbinolicus, AceP Methanosarcina acetivorans, AceP Methanosarcina barkeri, AceP Methanosarcina mazei, Methanospirillum hungateii, Anaeromyxobacter dehalogenans, AceP Methanococcoides burtonii, Methanococcus maripaludis, Sulfolobus acidocaldarius, Sulfolobus solfataricus P2, Picrophilus torridu, Thermoplasma acidophilum, Thermoplasma volcanium GSS1. [file 1471-2180-10-62-S3.PDF]
