# Supplementary material for: Carbon-dependent control of electron transfer and central carbon pathway genes for methane biosynthesis in the Archaean, Methanosarcina acetivorans strain C2A
Source: BMC Microbiol. 2010 Feb 23;10:62. doi: 10.1186/1471-2180-10-62 (PMC2838876; doi:10.1186/1471-2180-10-62)
Supplement: Additional file 4 — Table S1. Oligonucleotides used in this study. Description: This table provides the nucleotide sequence of all oligonucleotides used for PCR-based experiments. [file 1471-2180-10-62-S4.PDF]

Table S1

Oligonucleotides used in this study.

Primer Name	Sequence	Primer Name	Sequence
MA0304Rtfor	cacggcttcctgacctca	MA2433Rtfor	Cgcagcaacataaccgga
MA0304Rtrev	atggtagctccggcaagg	MA2433Rtrev	Gagcttcaatcggtcgtcc
MA0306Rtfor	cctggatgatgaagacgca	MA2434Rtfor	Gaagaggcactgtaaccgga
MA0306Rtrev	caggtggactggcattgg	MA2434Rtrev	Ggtaggccactcgatggtaa
MA0526Rtfor	atatggcgcaggcaacc	MA2441Rtfor	Gcgcgagaggacaattga
MA0526Rtrev	ctcttccggacctcttcga	MA2441Rtrev	Cgatgccggagccattat
MA0658Rtfor	ccgaacaccagcatctcc	MA2868Rtfor	Cggtggaaggcacaagg
MA0658Rtrev	tccgtctccgtcagcatc	MA2868Rtrev	Acgaacagaagccggagg
MA0661Rtfor	ggtcgaacggaaccaggt	MA2878Rtfor	Ggaatgctctgcgacga
MA0661Rtrev	ccaccggaagatccacaa	MA2878Rtrev	Gcttatcgccaacacgga
MA0663Rtfor	cctgcctctgattaccacca	MA3126Rtfor	Tcttcgtaccagcgcaca
MA0663Rtrev	cggacttcggcacttctacc	MA3126Rtrev	Cagcctctcatcacgcct
MA0688Rtfor	agcaggtggcggtgtaa	MA3128Rtfor	Caggttcagcacactggca
MA0688Rtrev	tgcgcttgagaggacatc	MA3128Rtrev	Gccggataagagccttgttg
MA0833Rtfor	ctgtcacggctcggctt	MA3998Rtfor	Caacacggtcgcagatctc
MA0833Rtrev	cttccgcactccatacacgt	MA3998Rtrev	Ccaaggtggcggttacaa
MA0834Rtfor	cgcggttataaggagcagg	MA4153Rtfor	Gcgacgagatcttgagcatc
MA0834Rtrev	aaggcaggacaatggtgg	MA4153Rtrev	Tatgccttgccgttggtg
MA0975Rtfor	ccacaacctggaacctcc	MA4156Rtfor	Gacgttgacgaactggca
MA0975Rtrev	atcctgttctcctcgtccgt	MA4156Rtrev	Gatatgccgcgatcaggtag
MA0978Rtfor	accacacgaaccagagcct	MA4159Rtfor	Accgaaggccagatcgtg
MA0978Rtrev	gttgagatgtcgccgagg	MA4159Rtrev	Cgattgccacgagacctct
MA1141Rtfor	gatgagcagatcggtggag	MA4174Rtfor	Aaggtatcctggcagtgg
MA1141Rtrev	caacgcaggcaatacagg	MA4174Rtrev	Ggacagactctcgcacaa
MA1143Rtfor	ccgatggttgttgagcgt	MA4175Rtfor	Ctcgccacgctcatgaa
MA1143Rtrev	ggcaggaaccgtgatcat	MA4175Rtrev	Catccaggccacataagca
MA1146Rtfor	cacgccgcggatattatg	MA4178Rtfor	Gactgtgcagcttaccttgg
MA1146Rtrev	cgagtggcacagtcagcat	MA4178Rtrev	Cgtcacactccatggca
MA1148Rtfor	caggatctacttcggctgg	MA4190Rtfor	Ccggtagccatgaggagaa
MA1148Rtrev	ttccgcatcctcctcctc	MA4190Rtrev	Gaccgcttcgagacctcat
MA1495Rtfor	ctgccagaatccagttca	MA4237Rtfor	Agccggaacatcgtcctc
MA1495Rtrev	cgtatccgaagagcagtatg	MA4237Rtrev	Gcctgacctcagctgttcc
MA1504Rtfor	ggtccgacaaccgtctca	MA4566Rtfor	Gctgccttgccgatcata
MA1504Rtrev	ccaggtacgcaatgacca	MA4566Rtrev	Gcacgcaacaaccaaggt
MA1506Rtfor	ggtctggatggtgagcca	MA4572Rtfor	Gccggagcaagcataacc
MA1506Rtrev	cgcctggatggttgaaga	MA4572Rtrev	Agtggccggtggtgtattc
